# The correlation of body mass index with clinical factors in patients with first-episode depression

**DOI:** 10.3389/fpsyt.2022.938152

**Published:** 2022-08-31

**Authors:** Hu Jieqiong, Ji Yunxin, Dai Ni, Lin Chen, Chai Ying

**Affiliations:** Department of Psychosomatic Medicine, Ningbo First Hospital, Ningbo, Zhejiang, China

**Keywords:** depression, body mass index (BMI), underweight, HAMD scores, uric acid

## Abstract

Depression is one of the major disease burdens worldwide. Few studies have addressed body mass index (BMI) in Chinese depression patients. This current study aimed to investigate the BMI in patients with depression and the correlation with clinical factors. A total of 211 inpatients with first-episode depression were enrolled. General and clinical data were collected by standardized questionnaires and the levels of hemoglobin, fasting blood glucose, uric acid, and blood lipid were measured. In total, 24-item Hamilton Depression Scale (HAMD) and 14-item Hamilton Anxiety Scale (HAMA) were rated for all the patients. The BMI of 211 patients with depression was 37 (17.5%) in the underweight group, 117 (55.5%) in the normal-weight group, 43 (20.4%) in the overweight group, and 14 (6.6%) in the obesity group. Multivariate logistic analysis showed that uric acid was the only risk factor for BMI. The ordered logit model showed that the risk of elevated uric acid increased with BMI grade. And the risk of severe depression was significantly greater in patients with underweight than those in normal-weight. The level of uric acid in patients with first-episode depression is positively correlated with BMI, and the score of depressive symptoms is higher in patients with underweight.

## Introduction

Depression is one of the major disease burdens worldwide. According to the 2019 Global Burden of Disease Study (1), depression is one of the top ten disease burdens among people aged 10–49 years, which negatively affects quality of life and social function, and even leads to suicide. More than 44 million people had disability-adjusted life-years (DALYs) attributed to depression. The lifetime incidence of depression in China was about 7% (2). On the basis of the huge population, the social and medical burden should not be underestimated. According to recent studies, depression is associated with an increased risk of all-cause and CVD mortality in adults in China (3). A meta-analysis that included 293 studies from 35 countries confirmed that there is a highly significant association between depression and excess mortality at follow-up (4). Global BMI mortality collaboration published a meta-analysis of 239 prospective studies in four continents in the Lancet showed that the correlation curve between BMI and mortality is U-shaped, and underweight increases all-cause mortality to the same degree as obesity, while the mechanism is unclear (5). Weight change is a common symptom of depression. Previous studies have discussed the relationship between depression and obesity, and the results are inconsistent. This study is aimed to describe the relationship between body mass index (BMI) and depression in the hospitalized patients.

## Materials and methods

### Design and sample

We adopted a descriptive cross-sectional design for our study. By collecting detailed medical information from each participant and performing physical, laboratory, and imaging examinations, we consecutively included the hospitalized patients with the following criteria between January 2020 and August 2021. (1) The diagnosis of depression was verified by two experienced psychiatrists according to the Chinese version of the Structured Clinical Interview for DSM-V. (2) Aged 18–60 years, Han Chinese, primary school education or above; (3) No prior treatment with an antidepressant; (4) No drug/alcohol abuse or dependence; (5) No major medical abnormalities, namely, central nervous system diseases, acute, unstable or life-threatening medical illnesses (such as infection, cancer, and organ failure); and (6) no pregnancy or lactation.

The ethical approval was obtained from the Clinical Research Ethics Committee of Ningbo First Hospital (No.2021RS124) before the study began. The procedures of this study were explained by an experienced psychiatrist, and all the participants signed informed consent to participate in the study.

### Collection of general information

• We adopted a case record form which recorded information on age, gender, marriage, education level, smoking or not, age of onset, and duration of illness for all the participants. Body mass index (BMI) was calculated as body weight in kilograms divided by the square of height in meters (kg/m^2^). Body weight and height were measured with OMRON ultrasonic height and weight meter (OMRON HNH-318) which was accurate to 0.1 kg and 0.1 cm. Participants were measured in light indoor clothes and standing barefoot on the measuring instrument. According to the guidelines for prevention and control of overweight and obesity in adults in China (6), all the participants were classified as underweight (<18.5 kg/m^2^), normal-weight (18.5–23.99 kg/m^2^), overweight (24–27.99 kg/m^2^), and obesity (≥28 kg/m^2^).

###  Clinical measurements

In total, two raters were trained on the use of the following two scales simultaneously before the study. The measurements were performed blindly with high inter-rater reliability. The severity of depressive symptoms was assessed by the 24-item Hamilton Rating Scale for Depression (HAMD) (7), which was including 10 items that scored 0∼2 and 14 items that scored 0∼4. The Chinese-language version was validated in 1985 (8). All the participants were divided into groups with or without severe depressive symptoms by the cut-off point of 35. The severity of anxiety was quantified by the 14-item Hamilton Anxiety Rating Scale (HAMA) (9). The Chinese-language version was validated in 1986 (10). All the participants were divided into groups with or without severe anxiety according to the cut-off point of 29. HAMD item 3 was used to assess suicidal ideation (SI) (11). The item has the following alternative statements: 0 = absent, 1 = feels life is not worth living, 2 = wishes he/she were dead or any thoughts of the possible death of self, 3 = suicide ideas or gesture, and 4 = attempts at suicide (any serious attempt rates). In this study, according to the cut-off point of 3, participants were divided into groups with or without SI.

### Serum assays

Blood samples from patients were collected between 6 and 8 a.m. following an overnight fast, and then measured on the same day. Hemoglobin (Hgb), fasting blood glucose (Glu), uric acid (UA), and blood lipid including triglyceride (TG), total cholesterol (TC), high-density lipoprotein (HDL-C), and low-density lipoprotein (LDL-C) were measured at the clinical laboratory of the Ningbo First Hospital. Hyperuricemia was defined as a serum urate level > 420 mmol/L (7.1 mg/dl) for both men and women, as recommended by the Chinese Multi-Disciplinary Expert Task Force on Hyperuricemia and Its Related Diseases (12).

### Data analysis

Kolmogorov–Smirnov one-sample test was used to confirm the normality. All categorical variables were expressed as numbers and percentages, continuous variables with normal distribution were expressed as mean ± standard deviation (SD), and variables with non-normal distribution were expressed as median (quartile). Categorical variables were compared by the χ^2^ test. Continuous variables that conformed to normality and homogeneity of variance were detected by Analysis of Variance (ANOVA), meanwhile, those not conforming were detected by the Kruskal–Wallis rank test. *Post-hoc* analysis was performed for pairwise comparison after the ANOVA or the Kruskal–Wallis rank test. Then, univariate and multivariable logistic analyses were performed to explore the relationship between clinical factors and BMI groups. BMI was considered to be the dependent variable. Furthermore, ordinal logistic analysis was performed to explore the relationships between BMI groups and UA level, BMI groups, and HAMD score, respectively. Both UA and HAMD scores were categorized into 5 levels according to the 4 equal division points of 20, 40, 60, and 80%.

SPSS (version 25.0) was used to do all the statistical analyses. All *p*-values were two-tailed with a significance level set at 0.05.

## Results

### Correlation of body mass index and demographic and clinical measures

The final sample size of this study was 211 participants. Of all the 211 participants, 37 patients were underweight (17.5%), 117 patients were of normal weight (55.5%), 43 patients were overweight (20.4%), and 14 patients were obese (6.6%). There was no gender difference among groups (*p* = 0.134).

Demographic profile and clinical characteristics were shown in Table 1, while biochemical characteristics were shown in Table 2. χ^2^ test and ANOVA or the Kruskal–Wallis rank test showed that the differences in age (*p* = 0.035), marriage (*p* = 0.009), age of onset (*p* = 0.039), HAMD score (*p* = 0.031), incidence of severe depressive symptoms (*p* = 0.028), incidence of hyperuricemia (*p* = 0.001), Hgb (*p* = 0.012), Glu (*p* = 0.044), UA (*p* = 0.00), TG (*p* = 0.00), TC (*p* = 0.001), LDL-C (*p* = 0.000), and HDL-C (*p* = 0.005) were significantly among the BMI groups. While no significant differences in education (*p* = 0.095), duration (*p* = 0.837), HAMA score (*p* = 0.053), incidence of SI (*p* = 0.081), and incidence of severe anxiety (*p* = 0.078) in these four groups.

**TABLE 1:** Demographic profile, clinical characteristics of first-episode depression inpatients from the Department of Psychosomatic Medicine of Ningbo First Hospital in different BMI groups.

	Total	Underweight (BMI < 18.5)	Normal-weight (18.5 = BMI < 24)	Overweight (24 = BMI < 28)	Obesity (BMI = 28)	*P*-value
Patient (%) (%)	211	37 (17.5%)	117 (55.5%)	43 (20.4%)	14 (6.6%)	
Age (years)	40 (23,54)^a^	35 (24.5,52)^a^	40 (22,54)^a^	51 (27,57)^a^	25.5 (22,41)^a^	0.035
Sex (male,%)	59 (28.0%)	9 (24.3%)	28 (23.9%)	15 (34.9%)	7 (50%)	0.134
Education (%)						0.095
Primary school	41 (19.4%)	6 (16.2%)	22 (18.8%)	11 (25.6%)	2 (14.3%)	
High school	110 (52.1%)	20 (54.1%)	65 (55.6%)	22 (51.2%)	3 (21.4%)	
College and above	60 (28.4%)	11 (29.7%)	30 (25.6%)	10 (23.2%)	9 (64.3%)	
Marriage (%)						0.009
Unmarried	94 (44.5%)	17 (45.9%)	54 (45.9%)	12 (27.9%)	11 (78.6%)	
Married	117 (55.5%)	20 (54.1%)	63 (53.8%)	31 (72.1%)	3 (21.4%)	
Smoking (%)	9 (4.3%)	3 (8.1%)	3 (2.6%)	3 (7.0%)	0 (0.0%)	0.275
Age of onset (years)	38 (22,53)^a^	34 (23.5,51)^a^	38 (21,53)^a^	50 (26,55)^a^	24.5 (21,40.25)^a^	0.039
Duration (months)	12 (6,24)^a^	12 (10,24)^a^	12 (6.5, 24) ^a^	12 (6, 24)^a^	15 (4.5, 24)^a^	0.837
HAMD score	32 (26, 38)^a^	35 (30, 43.5) ^a^	31 (25,36)^a^	32 (26, 35)^a^	31.5 (25, 34.75)^a^	0.031
HAMA score	25 (21, 30)^a^	28 (22.5, 33)^a^	25 (21, 30)^a^	23 (20, 28)^a^	23.5 (18, 27.5)^a^	0.053
Suicide ideal (%)	72 (34.1%)	19 (51.4%)	33 (28.2%)	15 (34.9%)	5 (35.7%)	0.081
Severe depression (%)	60 (28.4%)	18 (48.6%)	29 (24.8%)	10 (23.3%)	3 (21.4%)	0.028
Sever anxiety (%)	58 (27.5%)	16 (43.2%)	31 (26.5%)	9 (20.9%)	2 (14.3%)	0.078

BMI: body mass index; a: Continuous variable of normal distribution, expressed by X±S; b: Continuous variable of non-normal distribution, expressed by M (Q1, Q3).

**TABLE 2:** Biochemical characteristics of first-episode depression inpatients from the Department of Psychosomatic Medicine of Ningbo First Hospital in different BMI groups.

	Total	Underweight (BMI < 18.5)	Normal-weight (18.5 = BMI < 24)	Overweight (24 = BMI < 28)	Obesity (BMI = 28)	*P*-value
Hgb (g/dL)	128 (120,137)^a^	124 (114.5, 131.5)^a^	128 (120, 136)^a^	130 (122, 141)^a^	137 (125.5, 162.75)^a^	0.012
Glu (mmol/L)	4.78 (4.48,5.03)^a^	4.62 (4.42,4.85) ^a^	4.47 (4.45, 5.01)^a^	4.9 (4.59, 5.2)^a^	4.85 (4.43,5.15)^a^	0.044
UA (ummol/L)	309.87 ± 86.17^b^	273.77 ± 69.66^b^	305.17 ± 80.74^b^	321.32 ± 98.51^b^	409.39 ± 102.25^b^	0.00
Hyperuricemia	20 (9.5%)	1 (2.7%)	7 (6%)	6 (14%)	6 (42.9%)	0.001
TG (mmol/L)	1.1 (0.81,1.54)^a^	0.83 (0.63,1.06)^a^	1.10 (0.78, 1.43)^a^	1.34 (1.07, 1.73)^a^	1.2 (0.95, 1.81)^a^	0.00
TC (mmol/L)	4.58 ± 0.92^b^	4.09 ± 0.86^b^	4.607 ± 0.82^b^	4.88 ± 1.03^b^	4.63 ± 1.01^b^	0.001
LDL-C (mmol/L)	2.98 ± 0.70^b^	2.51 ± 0.62^b^	3.0 ± 0.589^b^	3.26 ± 0.788^b^	3.167 ± 0.85^b^	0.000
HDL-C (mmol/L)	1.23 (1.07,1.37)^a^	1.3 (1.19, 1.44)^a^	1.24 (1.10, 1.39)^a^	1.18 (1.02, 1.33)^a^	1.03 (0.89, 1.21)^a^	0.005

UA: uric acid; TG: triglyceride; TC:total cholesterol; LDL: low-density lipoprotein; HDL: high-density lipoprotein; a: Continuous variable of normal distribution, expressed by X ± S; b: Continuous variable of non-normal distribution, expressed by M (Q1, Q3).

Furthermore, *post-hoc* analysis found the incidence of unmarried in the obesity group was greater than that in the other three groups, including the underweight group (*p* = 0.037), the normal-weight group (*p* = 0.022), and the overweight group (*p* = 0.001). While the incidence of unmarried in the normal-weight group was higher than that in the overweight group (*p* = 0.038). The level of HAMD in the underweight group was higher than that in the normal-weight group (*p* = 0.027). The incidence of severe depressive symptoms in the underweight group was greater than the normal weight group (*p* = 0.006) and the overweight group (*p* = 0.018). However, there was no significant difference in the incidence of severe depressive symptoms between the underweight group and the obesity group (*p* > 0.05). The underweight group had a lower level in Hgb compared with the obesity group (*p* = 0.011) and a lower level in Glu (*p* = 0.028) compared with the overweight group. The levels of UA and the incidence of hyperuricemia in the obesity group were higher than those in the normal-weight group (*p* = 0.013; *P* = 0.001) and the underweight group (*p* = 0.001; *p* = 0.001). The level of TG in the underweight group was lower than that in the normal-weight group (*p* = 0.029), the overweight group (*p* = 0.000), and the obesity group (*p* = 0.015). While the levels of TG in the overweight group were higher than those in the normal-weight group (*p* = 0.011). The level of TC in the underweight group was lower than the normal-weight group (*p* = 0.014) and the overweight group (*p* = 0.002). The level of LDL-C in the underweight group was lower than the normal-weight group (*p* = 0.001) and the overweight group (*p* = 0.000). The level of HDL-C in the underweight group was higher than the obesity group (*p* = 0.009).

Then, variables with significant differences in *post-hoc* analysis were entered into univariate ordinal logistic regression analysis and the result showed that all the variables, except age, marriage, and age of onset, had a significant effect on BMI. These significant variables were further entered into multivariate ordinal logistic regression analysis and found that UA (OR = 1.004, 95% CI: 1.001–1.008, *p* = 0.019) was the only variable that statistically predicted BMI (Shown in Table 3).

**TABLE 3:** Univariate and multivariate ordinal logistic regression analysis of difference of clinical factors between BMI groups.

	Univariate model				Multivariable model			
	*P*-value	OR	95% CI		*P*-value	OR	95% CI	
			Lower bound	Upper bound			Lower bound	Upper bound
Severe depression	0.014	2.100	1.164	3.788	0.921	1.049	0.410	2.682
HAMD	0.005	0.963	0.963	0.939	0.503	0.981	0.928	1.037
Hgb	0.001	1.031	1.013	1.049	0.095	1.017	0.997	1.037
UA	0.000	1.007	1.004	1.010	0.019	1.004	1.001	1.008
Hgb	0.020	1.555	1.072	2.255	0.160	1.358	0.886	2.083
TG	0.014	1.582	1.096	2.286	0.955	1.012	0.678	1.508
TC	0.000	1.688	1.261	2.259	0.918	0.918	0.181	4.662
HDL-C	0.002	0.257	0.107	0.620	0.151	0.284	0.051	1.586
LDL-C	0.000	2.631	1.769	3.913	0.293	2.748	0.417	18.108
Marriage	0.808	0.938	0.557	1.579				
Age	0.320	1.009	0.991	1.027				
Age of onset	0.304	1.009	0.992	1.027				

### The association between uric acid and body mass index

The impact of BMI on UA levels was explored by ordinal logistic regression analysis (Shown in Table 4). The UA level was set as a dependent variable, and categorized into 5 levels according to quintile. BMI levels (underweight, normal-weight, overweight, and obesity) were considered as independent variables. Set the normal-weight group as the reference, the risks of uric acid levels increased were 0.42 (95% CI: 0.219–0.805, *p* = 0.009) in the underweight group, 1.534 (95% CI: 0.807–2.917, *p* = 0.192) in the overweight group, and 5.153 (95% CI: 1.847 ∼ 14.381, *p* = 0.002) in the obesity group.

**TABLE 4:** Ordinal logistic regression analysis of relationship between UA levels in normal-weight group and those in underweight group, overweight group and obesity group.

	OR	95%CI		*P*-value
		Lower bound	Upper bound	
Normal-weight (18.5 = BMI < 24)	1	.	.	.
Under-weight (BMI < 18.5)	0.420	0.219	0.805	0.009
Overweight (24 = BMI < 28)	1.534	0.807	2.917	0.192
Obesity (BMI = 28)	5.153	1.847	14.381	0.002

### The association between the Hamilton Depression Scale and body mass index

The relationship between BMI and HAMD scores was explored by ordinal logistic regression analysis (Shown in Table 5). Set the HAMD scores as a dependent variable, which were categorized into 5 levels according to quintile. BMI levels (underweight, normal-weight, overweight, and obesity) were considered as independent variables. Set the normal-weight group as the reference, the risks of HAMD level increased were 2.508 (95%CI: 1.269 ∼ 4.958, *p* = 0.008) in the underweight group, 0.994 (95%CI: 0.539 ∼ 1.833, *p* = 0.994) in the overweight group, and 1.136 (95%CI: 0.428 ∼ 3.012, *p* = 0.798) in the obesity group.

**TABLE 5:** Ordinal logistic regression analysis of relationship between HAMD levels in normal-weight group and those in underweight group, overweight group and obesity group.

	OR	95%CI		*P*-value
		Lower bound	Upper bound	
Normal-weight (18.5 = BMI < 24)	1	.	.	.
Under-weight (BMI < 18.5)	2.508	1.269	4.958	0.008
Overweight (24 = BMI < 28)	0.994	0.539	1.833	0.994
Obesity (BMI = 28)	1.136	0.428	3.012	0.798

## Discussion

The major findings of this exploratory cross-sectional clinical study showed that among 211 Chinese Han patients with first-episode depressive disorder, the proportion of underweight, normal-weight, overweight, and obesity were 17.5, 55.5, 20.4, 6.6%, respectively. UA was positively associated with body weight. The risk of elevated UA was increased with the increased BMI.

In this study, the proportion of underweight was 17.5%, higher than the general Chinese population, while the proportion of overweight (20.4%) and obesity (6.6%) in the general Chinese population were similar to our study (13). A survey of patients with MDD in China found that 9.9% of the patients were underweight, 27.7% were overweight and 7.4% were obese (14). Compared with the previous studies in Western Countries, the proportion of underweight in our sample was high and the proportion of overweight and obesity was low. A survey of patients with chronic or recurrent MDD in the United States showed that 1.7% of the patients were underweight, 28.8% were overweight, and 46.1% were obese (15). Another survey of patients with MDD in Europe showed that 48% of the patients were normal-weight, 31% were overweight, and 21% were obese, which grouped normal-weight and underweight individuals together because the number of underweight individuals was small (16). The main reason for these differences may ethnic factors. The proportion of obesity in the general American adult was 46.5%, of which 7.6% were severe obesity (17), while the proportion of obesity in the general Chinese adult was only 5.2% (18). Additional, long- term use of antidepressants may lead to weight gain (19, 20). Unlike most previous studies, we recruited first-episode patients who had no previous antidepressant treatment, thus avoiding the effects of the medication on BMI. Previous studies also found that the BMI in patients with depression increased as the duration of illness went longer because of the continuous reductions in physical activities and energy consumption (16). The median disease course in this study was 12 months which was relatively shorter than in the previous studies.

This study observed that UA was the only independent risk factor related to BMI among all clinical indicators of depression patients. Compared with the normal-weight group, the underweight group had a lower risk of increased uric acid, while the risk of increased uric acid was significantly higher in the overweight and obesity groups. A study of individuals from the Global Urate Genetics Consortium showed that BMI was positively associated with serum uric acid level which was consistent with the results of this study (21). We also found that the proportion of hyperuricemia in underweight, normal weight, and overweight patients were 2.7, 6, and 14%, respectively, lower than in the corresponding BMI groups in the general Chinese population, while the proportion of hyperuricemia in obesity (42.9%) was similar. A meta-analysis included 14 studies showed that participants with MDD had lower levels of uric acid as compared with the healthy controls, which may verify our results (22). Previous studies have found that uric acid, as the final product of the purine energy system, may affect the occurrence and development of a variety of psychiatric diseases (23). It is a major oxidative stress inducer and also a potent antioxidant, with more than 50% plasma antioxidant capacity provided by uric acid. A meta-analysis showed that participants with MDD had lower levels of uric acid, furthermore, antidepressant treatment seems to influence this association, which may indicate that the findings supported the hypothesis that uric acid levels may represent a state marker of MDD (22). Another large-sample case-control study showed that uric acid is lower in participants with current MDD in comparison to controls, which was independent of antidepressant use. In addition, the uric acid levels of the participants with remitted MDD did not differ from the controls (24). These findings suggested lower uric acid is a status marker, not a trait characteristic of participants with MDD. On the other hand, along with being underweight, hypouricemia was also considered to be one of the markers of malnutrition. The incidence of underweight was higher in patients with depression in this survey than in the general population in previous studies, which may be due to reduced eating and behavioral disorders. In this study, the underweight group had lower hemoglobin levels, fasting blood glucose levels, uric acid levels, triglyceride levels, total cholesterol levels, and LDL levels than one or more of the other BMI categories. The result suggests that the nutritional status of patients underweight is poor, which may aggravate dizziness, fatigue, and other physical discomforts.

We also explored the relationship between BMI and severity of depression (HAMD score), and found that the risk of severe depression was significantly greater in patients with underweight than those in normal-weight. These results suggested that the depressive symptoms of underweight patients may be more severe than those of normal-weight patients, while no significant differences in HAMD scores between the overweight or obesity groups compared with the normal-weight group. Previous studies in the United States and Europe had reported that the severity of depressive symptoms in patients with MDD was positively correlated with BMI (16, 25). A survey of patients with first-episode MDD in China found that HAMD score increased the risk of weight gain across two specific score ranges, and had no clear linear or no-linear correlation between HAMD score and BMI (26). Studies on the general population reported a U-shaped relationship between depression and BMI in Korean adults (27). And depressive symptoms decreased as BMI increased in the Chinese adults aged 45 years and older, with no gender difference, which indicated that there was an inverse association between BMI and depressive symptoms among the Chinese population, and supports the “jolly fat” hypothesis in China (28). Furthermore, being underweight was associated with a higher prevalence of depressive symptoms in the Chinese population, especially in women and the young (29, 30). The result of this study showed that the depressive symptoms in the underweight group were severe than in the normal-weight group, meanwhile, the depressive symptoms in the overweight group or the obesity group were not differ from that in the normal-weight group. This inconsistency may be explained by the heterogeneity in studies including inclusion criteria, ethnicity, medication application, and course of the disease. These results suggested that depression patients with underweight also have important research value, as well as obesity, especially in China.

There were some limitations in this article. This study was a cross-sectional study and a causal relationship between BMI and related risk factors could not be demonstrated. The small sample size and the gender imbalance may lead to relative bias in statistical analysis. Although none of the participants in this survey developed COVID-19 infection, factors such as COVID-19 fears, reduced incomes, and limited travel might affect the incidence and severity of depression in the epidemic situation, and the failure to include COVID-19 as a confounding factor was one of the limitations of this study too. In future studies, we will expand the sample size, enroll healthy participants, and consider COVID-19 status as a confounding variable that may provide more research results.

## Data availability statement

The raw data supporting the conclusions of this article will be made available by the authors, without undue reservation.

## Ethics statement

The studies involving human participants were reviewed and approved by the Clinical Research Ethics Committee of Ningbo First Hospital. No. 2021RS124. The patients/participants provided their written informed consent to participate in this study.

## Author contributions

HJ: conceptualization, methodology, software, investigation, formal analysis, and writing – original draft. JY: conceptualization, funding acquisition, resources, supervision, and writing – review. DN: data curation, visualization, and investigation. LC: supervision and investigation. CY: resources and validation. All authors contributed to the article and approved the submitted version.
